# COL5A1 Serves as a Biomarker of Tumor Progression and Poor Prognosis and May Be a Potential Therapeutic Target in Gliomas

**DOI:** 10.3389/fonc.2021.752694

**Published:** 2021-11-16

**Authors:** Sujie Gu, Zesheng Peng, Yuxi Wu, Yihao Wang, Deqiang Lei, Xiaobing Jiang, Hongyang Zhao, Peng Fu

**Affiliations:** ^1^ Department of Neurosurgery, Union Hospital, Tongji Medical College, Huazhong University of Science and Technology, Wuhan, China; ^2^ Department of Neurosurgery General Hospital of The Yangtze River Shipping, Wuhan, China

**Keywords:** COL5A1, glioma, temozolomide resistance, tumor-infiltrating immune cells (TIICs), tumor progression, bioinformatics analysis

## Abstract

Glioma is the most common malignancy of the central nervous system. Although advances in surgical resection, adjuvant radiotherapy, and chemotherapy have been achieved in the last decades, the prognosis of gliomas is still dismal. COL5A1 is one of the collagen members with minor content but prominent functions. The present study examined the biological functions, prognostic value, and gene-associated tumor-infiltrating immune cells of COL5A1 through experiments and bioinformatics analysis. We found that the overexpression of COL5A1 was positively correlated with the increasing tumor malignancies and indicated poor prognosis in gliomas. Moreover, downregulation of COL5A1 could inhibit proliferation and migration of glioma cells and enhance their temozolomide sensitivities *in vitro*. Further bioinformatic analysis revealed that COL5A1 and its co-expressed genes participated in a number of pathways and biological processes involved in glioma progression. Finally, we evaluated the tumor-infiltrating immune cells of gliomas depending on COL5A1 and found that the percentages of the dendritic cells, which were known as the central mediator of tumor microenvironment in gliomas, were positively associated with the expression levels of COL5A1. Taken together, COL5A1 is an important biomarker and potential therapeutic target of gliomas.

## Introduction

Glioma is the most common malignancy in the brain, accounting for more than 70% malignancies of the entire central nervous system (1). Although surgical resection followed by radiotherapy and chemotherapy has become the current standard care for glioma, its prognosis is still dismal, especially for glioblastoma (GBM), of which the 5-year survival rate is less than 5% (2). Due to the characteristics of invasive growth and tumor heterogeneity, complete cure of gliomas is usually impossible. Since a majority of low-grade gliomas, during treatment, convert to higher grades or even evolve in secondary GBMs, tumor progression is a thorny problem in the treatment of gliomas. Temozolomide (TMZ) is an oral antitumor drug approved by the US Food and Drug Administration (FDA) for the treatment of glioma. As a DNA-alkylating agent, TMZ is known to induce cell-cycle arrest at the G2/M phase and eventually lead to apoptosis (3). Unfortunately, at least 50% of gliomas do not respond to TMZ, of which some patients are intrinsically positive for resistance-related genes, such as p53 mutation, homeostatic iron regulator (HFE) mutation, and O^6^-methylguanine-DNA alkyltransferase (MGMT) promoter unmethylation; the others acquire the resistance after TMZ administration (4). Therefore, it is crucial to pursuing new targets of gliomas concerning tumor progression and TMZ chemoresistance for improving prognosis of gliomas.

Collagen type V alpha 1 chain (COL5A1), located at the long (q) arm of chromosome 9, encodes collagen type V, which may be a structurally minor player but functionally prominent in the collagen hierarchy (5). Collagen type V plays an important role in regulating fiber diameter and assembly of collagen fibers (6). COL5A1 was reported to promote proliferation and metastasis of multiple tumors, such as lung cancer, gastric cancer, breast cancer, and renal cell carcinoma (7–10). Moreover, a previous study found that the overexpression of COL5A1 was correlated with tumor-infiltrating immune cells (TIICs) and enhanced the paclitaxel resistance in ovarian cancer (11). However, the biological functions of COL5A1 in gliomas are still unclear.

In the present study, we investigated the expression levels of COL5A1 in gliomas with different grades or pathological types based on public data and clinical samples. Then, the prognostic value, co-expressed gene cluster and involved pathways, biological processes, and associated tumor-infiltrating immune cells in respect of COL5A1 were explored *via* bioinformatic analysis. Furthermore, cell experiments were conducted to examine the effects of downregulating COL5A1 in glioma cells on cell phenotypes such as proliferation, migration, and chemoresistance to TMZ.

## Material and Methods

### Cell Culture and Transfection

Human glioma cell lines of U87 and U251, purchased from the American Type Culture Collection (ATCC, Gaithersburg, MD, USA), were cultured in DMEM medium containing 10% fetal bovine serum and 1% penicillin and streptomycin in an incubator at 37°C with 5% CO_2_. The specific COL5A1 small interfering RNA (si-COL5A1, 5′-CGAGGGUGAGACCUAUUACUA-3′) and non-specific control siRNA (NC, 5′-UUCUCCGAACGUGUCACGUUU-3′) were synthesized by Wuhan Gene Create Corporation and transfected according to the manufacturer’s instructions. Three groups of glioma cells were included in this study, of which the blank group referred to cells not transfected, while the non-specific control (NC) and si-COL5A1 groups represented glioma cells transfected with non-specific control siRNA and si-COL5A1, respectively.

### RNA Extraction and Real-Time Polymerase Chain Reaction

Total RNA of cells was isolated with TRIzol reagent (15596-026, Ambion). After lysing for 5 min, trichloromethane was added into the mixed solution. Then, the supernatant was mixed with isopropyl alcohol and centrifuged for 10 min at 12,000 rpm and 4°C. Following washing with 75% alcohol for twice, the precipitated RNA was dissolved by DEPC-water and stored at -4°C. Reverse transcription was performed, and the synthesized cDNAs were amplified and quantified using the QuantStudio 6 PCR system (ABI). Glyceraldehyde 3-phosphate dehydrogenase (GAPDH) was used as an internal control. The primer sequences are listed as follows: GAPDH, forward, 5′-GGAAGCTTGTCATCAATGGAAATC-3′, reverse, 5′-TGATGACCCTTTTGGCTCCC-3′, COL5A1, forward, 5′-AGATGGCAAGTGGCACAGAAT-3′, reverse, 5′-GGTGGTCCGAGACAAAGAGC-3′. Calculation and quantification of gene expression were based on the 2^- ΔΔCt^ method.

### Protein Extraction and Western Blot Analysis

The proteins were isolated by RIPA lysis buffer (Beyotime, P0013B), and the concentrations were determined by the BCA detection kit (Beyotime, P0010). About 30 μg proteins was loaded and electrophoresed onto 8% SDS polyacrylamide gel (GE Healthcare Bioscience, 30166428) and then transferred to polyvinylidene fluoride (PVDF) membranes (Millipore, IPVH00010). The membranes were blocked with 5% non-fat milk for 60 min at 37°C and were then incubated with primary antibodies (COL5A1, 1:1,000, Abcepta, AP10487a, β-actin, 1:2,000, Servicebio, GB11001) overnight at 4°C, anti-rabbit secondary antibodies (1:5000, Servicebio, GB1213) at room temperature for 2 h. The detection of signal was conducted using Bryo ECL Kit (Beyotime, P0020) and the measurement of proteins’ expression was done by ImageJ (Version 1.8.0).

### Immunohistochemical Staining Analysis

Human glioma tissues were collected from patients that underwent craniotomy in Wuhan Union Hospital. The study was approved by the Ethics Committee of Wuhan Union Hospital, and written informed consent was obtained from these patients. A total of 18 glioma samples, including 6 WHO grade II, 6 grade III, and 6 grade IV, were used in this step. After fixing with 4% paraformaldehyde, embedding in paraffin, and slicing specimens into 5-μm-thick sections, sections were deparaffinized and dehydrated. After antigen retrieval and endogenous peroxidase destruction, sections were incubated with a primary antibody (COL5A1, 1:500) overnight and then incubated with a secondary antibody (1: 50, Beyotime, A0208). Sections were counterstained with hematoxylin after treating horseradish peroxidase and 3,3′-diaminobenzidine. Images were captured by an optical microscope, and ImageJ software was applied to count the targeted positive areas. The percentages of COL5A1-positive staining area were calculated using three different visual fields from each section at a magnification of ×400.

### Cell Proliferation and TMZ Sensitivity Detection

Cell Counting Kit-8 (CCK-8, Beyotime, C0038) was used to detect cell proliferation and TMZ sensitivity. After transfection for 48 h, 5,000 cells were seeded in 96-well plates to culture for 24, 48, and 72 h, then 10 μl of CCK-8 reagent was added and the relative numbers of cells were calculated based on absorbance of each well at a wavelength of 450 nm. For the drug sensitivity test of TMZ, a density of 10,000 cells were seeded in 96-well plates to culture for 12 h, then TMZ with gradient concentrations was added and the CCK-8 reagent was added to measure the relative cell concentrations after 48 h.

### Colony Formation Assay and Wound Healing Assay

Glioma cells were planted in six-well plates with a density of 1,000 cells per well. After culturing for 1 week, the cells were fixed with 4% paraformaldehyde and stained with 0.5% crystal violet. The numbers of colonies were counted by ImageJ. As for wound healing assay, 5*10^5^ cells were seeded in six-well plates, and a 1,000-μl sterile tip was used to scratch the wound when the cells reached 100% confluency. Then, the serum-free medium was used for subsequent cell culture to exclude the impact of cell proliferation. Pictures were obtained under an optical microscope at 0 and 24 h to calculate the healing percentage of the wound.

### Flow Cytometry

In this study, flow cytometry was used to detect cell cycle and apoptosis. For cell-cycle assays, U87 cells from all groups were harvested and washed with PBS three times. Then, 70% ethanol was used to fix the cells at 4°C for 30 min. After incubation with RNase (50 μg/ml) at 37°C for 30 min, cells were stained with propidium iodide (PI) (Sigma, P4170) in the dark and analyzed using a flow cytometer (Beckman, CytoFLEX). For apoptosis detection, cells were cultured with complete medium containing TMZ (50 μg/ml) for 48 h and then examined with Annexin V-FITC/PI detection kit (Elabscience, E-CK-A211) according to the manufacturer’s instruction. The % apoptosis was the sum of Annexin V-FITC+/PI- (early apoptosis) and Annexin V-FITC+/PI+ (late apoptosis) cells.

### Gene Expression Distribution and Prognostic Analysis Based on Samples From the CGGA and TCGA Databases

The RNA-seq data and clinical information of glioma samples were retrieved from The Cancer Genome Atlas (TCGA) database (https://xenabrowser.net/datapages/) (12) and Chinese Glioma Genome Atlas (CGGA) database (http://www.cgga.org.cn/download.jsp) (13). The gene expression distribution of COL5A1 among different grades and pathological types was analyzed and displayed by R software (Version 3.6.2) and “ggplot2” package. The prognostic value of COL5A1 was also examined and presented using R software and “survival” package.

### Co-Expressed Genes’ Prediction, Protein–Protein Interaction Network’s Construction, and Function Enrichment Analysis

The co-expressed genes of COL5A1 in GBM based on the expression profiles of TCGA GBM datasets were predicted by the LinkedOmics database (http://www.linkedomics.org/login.php) (14). The top 50 positive co-expressed genes and an equal number of negative co-expressed genes were selected for further analysis. The protein–protein interaction (PPI) network co-expressed genes were constructed using String Version 11.0 (https://string-db.org/) with a confidence of 0.7 and presented by Cytoscape (Version 3.6.1). Function annotation of Gene Ontology Biological Process (GO-BP), Gene Ontology Cellular Component (GO-CC), Gene Ontology Molecular Function (GO-MF), and Kyoto Encyclopedia of Genes and Genomes (KEGG) pathways were performed by means of DAVID Bioinformatics Resources 6.8 (15) and displayed by the “ggplot2” R package.

### Gene Set Enrichment Analysis in the GBM Cohort From TCGA

Expression profiles of 173 GBM samples were downloaded from TCGA database. KEGG and hallmark gene sets with different COL5A1 expressions were performed with Gene Set Enrichment Analysis (GSEA) 4.1 (http://www.broadinstitute.org/gsea/).

### Immune Infiltration Analysis

Tumor Immune Estimation Resource (TIMER) is a comprehensive database designed for the analysis of immune cell infiltration across different types of cancers (16). We analyzed the relationship between COL5A1 expression and the abundance of six immune infiltrating cells, including B cell, CD8+ T cell, CD4+ T cell, macrophage, neutrophil, and dendritic cell, using the TIMER “gene” module. Then, the influences of the correlated immune cells on prognosis of GBM and LGG were explored in the TIMER “survival” module.

### Statistical Analysis

GraphPad Prism (Version 8.4.2) was used to analyze all data that were expressed as the means ± standard deviation (SD). Two-tailed Student’s t test was employed to evaluate the difference between two groups; one-way ANOVA test was used to evaluate differences among three groups, and p < 0.05 was considered significant. All experiments were repeated at least three times, and representative results are shown.

## Results

### Overexpression of COL5A1 Is Correlated With Higher Tumor Grades and Malignancy Progression of Gliomas

**Figure 1:**
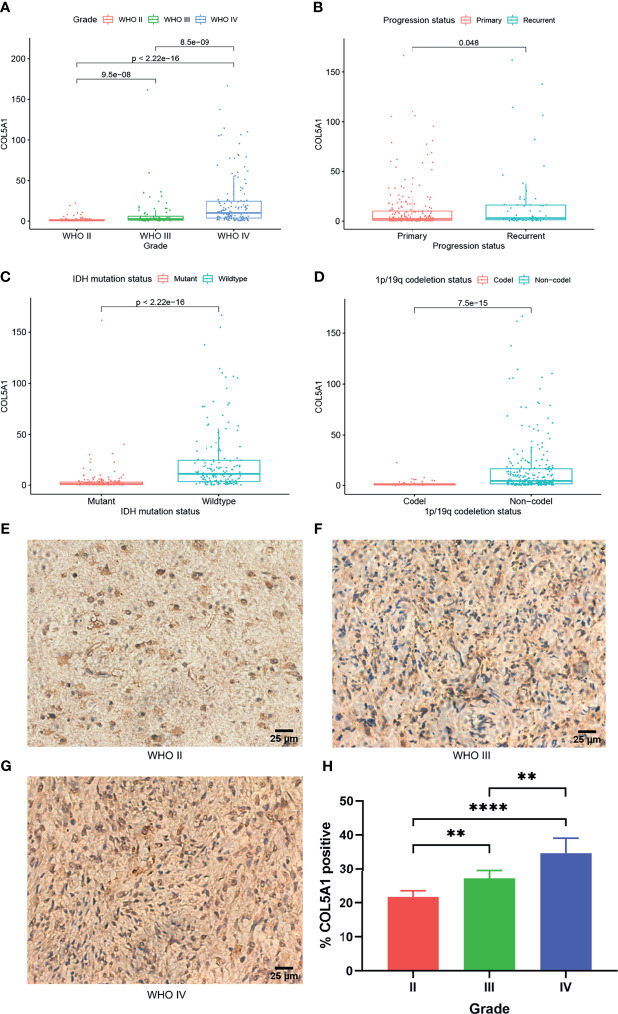
COL5A1 is correlated with multiple malignant features of gliomas. **(A)** Expression levels of COL5A1 in gliomas of different grades. **(B)** Expression levels of COL5A1 in primary and recurrent gliomas. **(C)** Expression levels of COL5A1 in gliomas with IDH mutant and wild types. **(D)** Expression levels of COL5A1 in gliomas with 1p/19q co-deletion and non-co-deletion. **(E–H)** Representative images of IHC staining of COL5A1 in different grades glioma tissues and the corresponding % positive area (**p < 0.01; ****p < 0.0001).

To investigate the expression distributions of COL5A1 among different tumor grades, pathological phenotypes, and tumor progression status, the expression profiles and clinical information of gliomas obtained from the CGGA database were integrated and analyzed. We found that the expression levels of COL5A1 increased along with tumor grades significantly (**Figure 1A**). Especially in the GBMs (WHO IV), COL5A1 was more highly expressed than the grade II and III gliomas dramatically (p < 0.001). The results of the immunohistochemical analysis based on glioma samples were also consistent with the trend above (**Figures 1E–H**). As shown in **Figure 1B**, the recurrent gliomas exhibited higher levels of COL5A1 than the primary ones (p < 0.05). We also evaluated the levels of COL5A1 in gliomas according to their isocitrate dehydrogenase (IDH) mutation and 1p/19q co-deletion status, which served as important molecular markers for identifying glioma subtypes. The results demonstrated that COL5A1 was obviously overexpressed in groups of IDH wild-type and 1p/19q non-co-deletion (p < 0.0001, **Figures 1C, D**).

### Overexpression of COL5A1 Indicates Dismal Prognosis in Gliomas and Correlates With Pathways in Regard to Tumor Progression

**Figure 2:**
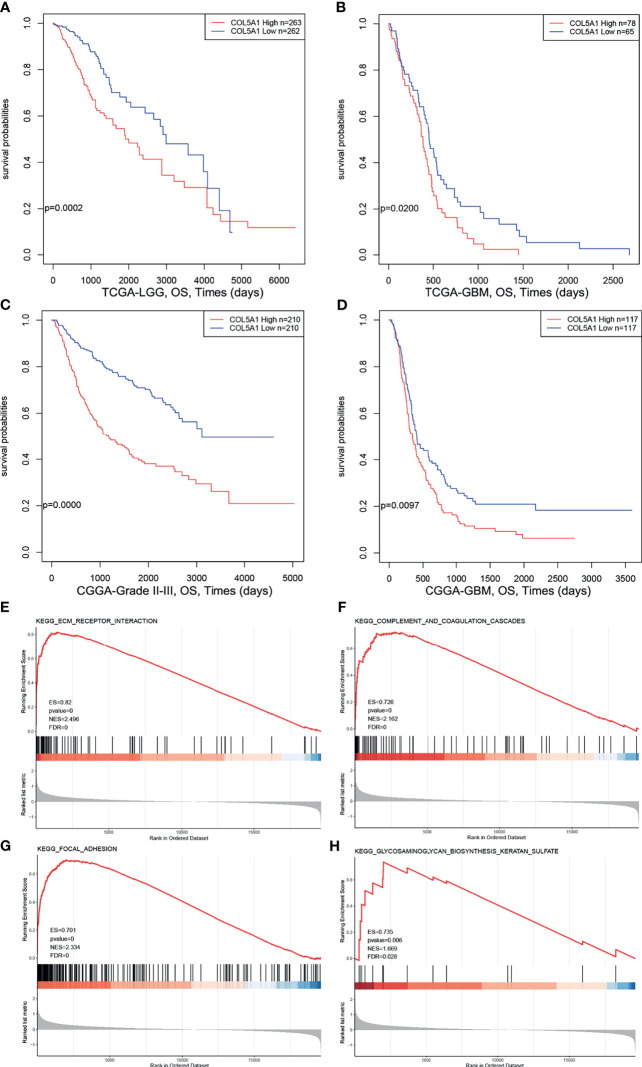
High expression levels of COL5A1 indicates poor prognosis and correlates with KEGG pathways related to tumor progression. **(A–D)** Survival analysis based on expression levels of COL5A1. **(E–H)** The top four positive KEGG pathways of GSEA based on COL5A1 (ES, enrichment score; NES, normalized enrichment score; FDR, false discovery rate).

In order to explore the prognostic value of COL5A1 in gliomas, expression profiles and clinical information of glioma samples from the CGGA and TCGA databases were retrieved for prognosis analysis. As shown in **Figures 2A, B**, the overall survivals of patients with lower expression of COL5A1 in the TCGA LGG and TCGA GBM datasets were longer than those of higher groups. Similar trends were observed in CGGA glioma datasets (**Figures 2C, D**), of which patients were divided into grade II–III and GBM groups. To assess the biological functions of COL5A1 in gliomas, we performed gene set enrichment analysis (GSEA) based on mRNA expression profiles of 173 glioblastomas from the TCGA database. Intriguingly, a high expression of COL5A1 was mainly enriched in the KEGG pathways of the ECM receptor interaction, complement and coagulation cascades, focal adhesion, and glycosaminoglycan biosynthesis keratan sulfate, which were all related to progression or chemoresistance of glioma cells (**Figures 2E–H**).

### COL5A1 Is Associated With Other Genes Related to Tumor Progression

**Figure 3:**
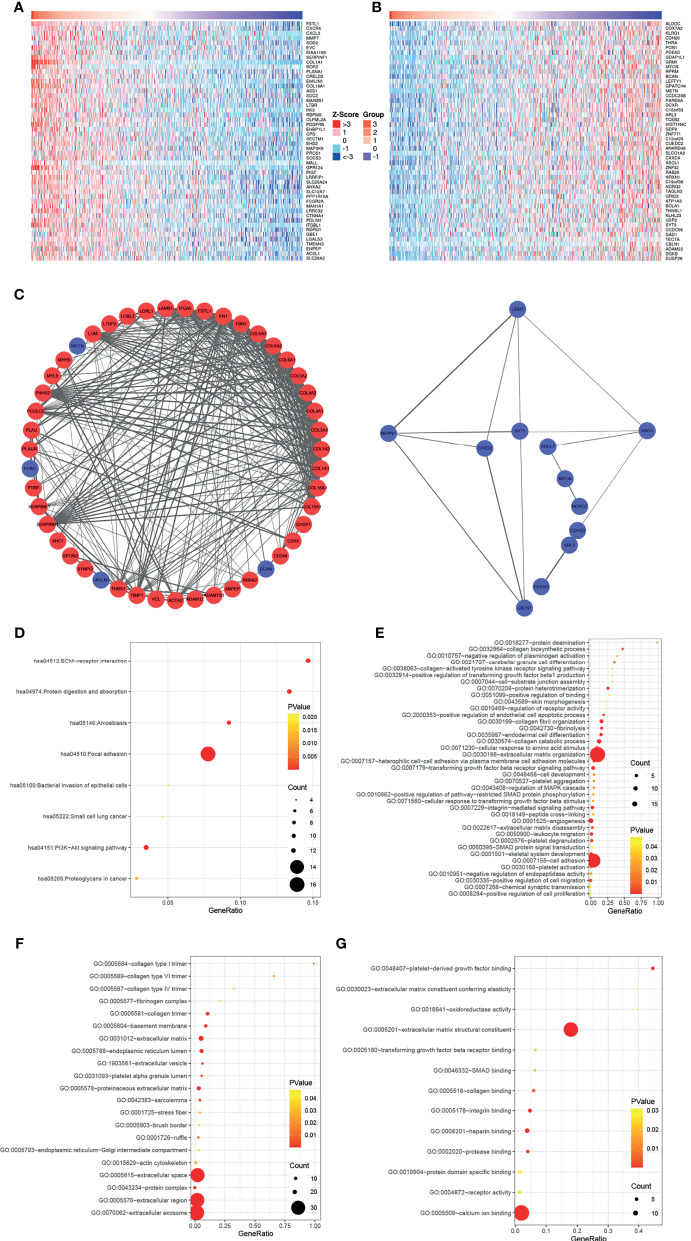
COL5A1 co-expressed genes and function enrichment analysis based on them. **(A, B)** Heatmaps showing the top 50 genes positively or negatively correlated with COL5A1; red and blue represent positively and negatively associated genes, respectively. **(C)** The networks of genes positively and negatively correlated with COL5A1. **(D–G)**. Functional enrichment analysis of the selected 100 genes; bubble plots represent significantly enriched terms in KEGG pathways and GO categories (p < 0.05) (BP, biological processes; CC, cell component; MF, molecular functions).

To further elucidate the biological functions of COL5A1, the function module of LinkedOmics was used to screen genes correlated with COL5A1 based on the expression profiles of TCGA GBM datasets. The heatmaps in **Figures 3A, B** presented the top 50 genes positively, and an equal number of genes negatively correlated with COL5A1. Genes positively related to COL5A1 included oncogenic genes like FSTL3, CXC chemokine family members (CXCR4, CXCL3), and MMP7. On the other hand, the negatively correlated genes included antitumor genes such as ALDOC, COX7A2, and KLRG1. The PPI networks of the co-expressed genes were constructed and visualized as **Figure 3C**, which suggested collagen members, such as COL8A1, COL1A1, and COL1A2, were the main connected components with COL5A1. Besides, LUM, P4HA2, PCOLCE, and SERPINH1 were main proteins interacting with the collagen members above. As for the negatively correlated proteins, GRM1, NRXN1, and GAD1 were the hub proteins. Interestingly, the function enrichment analysis of genes co-expressed with COL5A1 concluded a number of significant KEGG pathways and GO categories (p < 0.05) participating in the malignant behavior of proliferation, invasion, and migration in cancers (**Figures 3D–G**). For instance, the significantly enriched KEGG pathways consisted of ECM–receptor interaction, focal adhesion, protein digestion and absorption, and so on (**Figure 3D**). The enriched GO BPs included positive regulation of transforming growth factor beta 1 production (GO: 0032914), transforming growth factor beta receptor signaling pathway (GO: 0007179), extracellular matrix organization (GO: 0030198), positive regulation of cell migration (GO: 0030335), and positive regulation of cell proliferation (GO: 0008284) (**Figure 3E**). Similarly, extracellular matrix (GO: 0031012), collagen trimer (GO: 0005581), actin cytoskeleton (GO: 0015629), extracellular matrix structural constituent (GO: 0005201), and extracellular matrix constituent conferring elasticity (GO: 0030023) were included in the enriched terms of GO CC and GO MF (**Figures 3F–G**).

### Knockdown of COL5A1 Inhibits Proliferation and Migration and Enhances TMZ Sensitivity of Glioma Cells *In Vitro*

**Figure 4:**
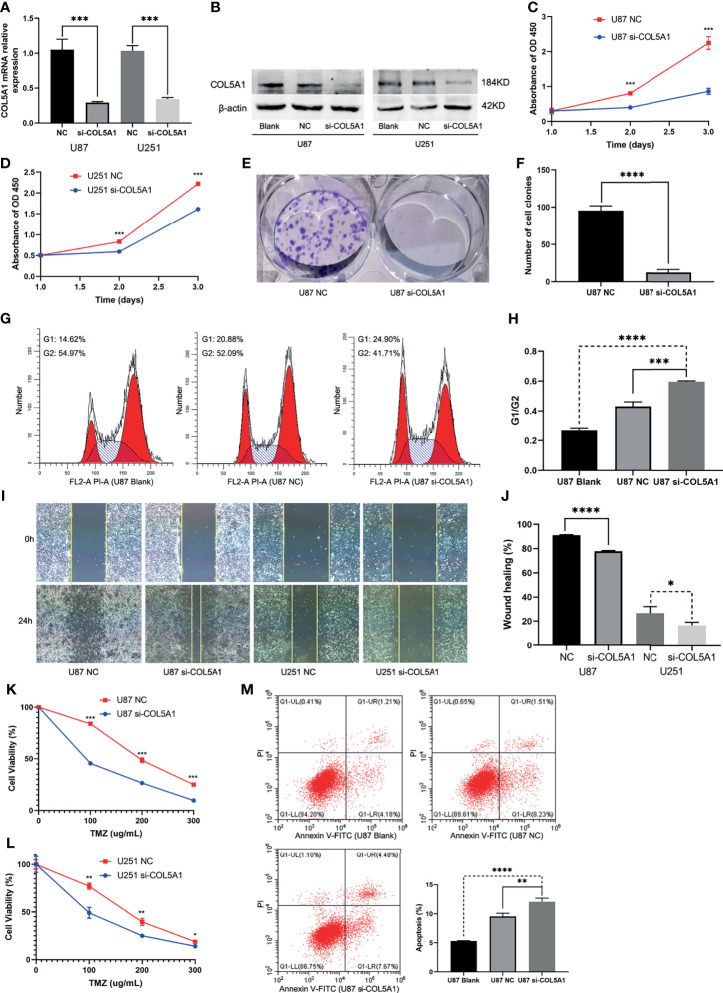
Knockdown of COL5A1 inhibits proliferation, migration, and TMZ resistance of glioma cells *in vitro*. **(A, B)** The relative expressions of COL5A1 in U251 and U87 cells were analyzed by qRT-PCR and Western blot after transfected with si-COL5A1. **(C, D)** The proliferation ability was determined using the CCK-8 assay following 3 days of culture. **(E, F)** The long-term cell viability was evaluated using the colony formation assay. **(G, H)** The cell-cycle phase of U87 cells was analyzed by flow cytometry. **(I, J)** Wound healing assay was performed to explore the cell’s migration ability. **(K, L)** Knockdown of COL5A1 could sensitize glioma cells to temozolomide. **(M)** Knockdown of COL5A1-sensitized tumor to TMZ chemotherapy by promoting apoptosis (*p < 0.05; **p < 0.01; ***p < 0.001, ****p < 0.0001).

The specific siRNA (si-COL5A1) was transfected into U87 and U251 cells to knock down COL5A1 expression, and the knockdown efficiency was validated by real-time polymerase chain reaction (RT-PCR) and Western blotting (**Figures 4A, B**). **Figures 4C, D** showed that knockdown of COL5A1 inhibited cell proliferation of glioma cells. Especially for U87 cells, the decrease of COL5A1 resulted in a more than three-fold proliferation reduction on the third day of CCK-8 assays (p < 0.001). Moreover, the results of colony formation assay suggested that the long-term proliferation ability was weakened obviously (p < 0.0001) by COL5A1 knockdown (**Figures 4E, F**). To clarify the mechanism of COL5A1 knockdown inhibiting glioma cell proliferation, we performed flow cytometry and detected the cell cycles of U87 cells with or without siRNA intervention. Compared with the blank and NC groups, higher ratios of G1/G2 were observed in the si-COL5A1 group (p < 0.001, **Figures 4G, H**), suggesting that decreased COL5A1’s inhibition of tumor proliferation may be through blocking the cell cycle in the G1 phase. We also performed wound healing assays to explore the effect of COL5A1 knockdown on the cell’s migration ability. As presented in **Figures 4I, J**, the migration abilities of U87 and U251 cells, especially the U87 cells (p < 0.0001), decreased dramatically when COL5A1 was downregulated. In the drug toxicity assays of TMZ (**Figures 4K, L**), cell viabilities of U87 and U251 in si-COL5A1 groups were all worse than those of the NC groups when exposed to gradient concentrations of TMZ from 100 to 300 μg/ml (p < 0.05), indicating that downregulation of COL5A1 could sensitize glioma cells to temozolomide obviously. We also used flow cytometry to further illustrate the mechanism of TMZ sensitivity enhancement induced by COL5A1 knockdown. After exposure to 50 μg/ml TMZ for 2 days, the percentage of apoptotic cells in the si-COL5A1 group was higher than both of the blank and NC groups (p < 0.01, **Figure 4M**), suggesting that COL5A1 knockdown may sensitized glioma cells to TMZ *via* promoting apoptosis.

### Overexpression of COL5A1 Is Positively Correlated With High Proportions of Tumor-Infiltrating Dendritic Cells in Gliomas

**Figure 5:**
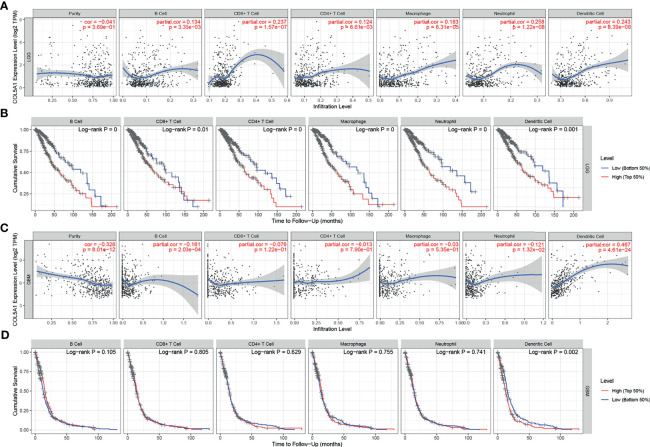
Analysis of tumor-infiltrating immune cells (TIICs) based on COL5A1. **(A, C)** The correlation analysis of COL5A1 and TIICs in LGG and GBM. **(B, D)** Survival analysis based on TIICs in LGG and GBM. (cor, correlation coefficient; partial.cor, partial correlation coefficient).

The TIMER database was used to evaluate the relationships between the expression levels of COL5A1 and the abundance of TIICs in gliomas based on the TCGA database. In LGGs, the COL5A1 expression level was positively correlated with all the six kinds of TIICs (partial.cor > 0, p < 0.01, **Figure 5A**), while in GBMs, its correlations with only B cell (partial.cor < 0, p < 0.001), neutrophil (partial.cor < 0, p < 0.05), and dendritic cell (partial.cor > 0, p < 0.0001) appeared to be significant (**Figure 5C**). Then, we performed prognosis analysis depending on each kind of TIIC using the TIMER “survival” module. Interestingly, we found that the high levels of the six TIICs all indicated poor prognosis in LGGs (p < 0.01, **Figure 5B**). However, only high levels of tumor-infiltrating dendritic cells (TIDCs) led to poor prognosis in GBMs (p < 0.01, **Figure 5D**). The above results demonstrated that (i) overexpression of COL5A1 was positively associated with larger proportions of TIDCs in all grades of gliomas and (ii) a high level of TIDCs indicated poor prognosis in gliomas.

## Discussion

As the most abundant proteins in mammals, collagens are deposited in the extracellular matrix (ECM), contributing to mechanical properties, organization, and shape of tissues (17). During the occurrence and development of cancers, the ECM undergoes structural changes, in which the content and distribution of collagens are modified to coordinate with cancers’ biological properties. Collagens in turn interact with cancer cells *via* several receptor families and regulate their proliferation, migration, and metastasis (18). COL5A1, considered as a structurally minor player playing important roles in the collagen hierarchy, was reported to promote progression and chemoresistance and affect tumor-infiltrating immune cells in multiple cancers (7–11). In our study, the role of COL5A1 in gliomas was comprehensively explored based on biological experiments and bioinformatics analysis for the first time.

We observed significant differences in the incidence of COL5A1 expression between different grades and different pathologic types of gliomas: (i) high-grade gliomas expressed significantly higher levels of COL5A1 compared with low-grade gliomas; (ii) recurrent gliomas expressed a slightly more amount of COL5A1 than the primary; (iii) IDH wild-type gliomas had a higher COL5A1 expression than the IDH mutation type; and (iv) gliomas with 1p/19q co-deletion expressed lower levels of COL5A1 than the 1p/19q non-co-deletion type. A previous study by Deng et al. revealed that COL5A1 positively correlated with latent transforming growth factor-beta binding protein 1 (LYBP1), and they might work together to promote tumor progression *via* regulation of extracellular matrix (ECM) in GBM (19). Another study reported that the expression level of COL5A1 in recurrent LGG was significantly higher than the primary LGG (20). IDH mutation and 1p/19q co-deletion are important biomarkers and serve as basis for identifying glioma molecular subtypes (21, 22). IDH wild-type tumors have worse prognosis and thus are regarded as unrecognized glioblastomas (23). According to the 4th edition of the WHO classification, glioblastomas were separated into two major types based on mutations in the IDH 1 or 2 genes, with IDH wild-type glioblastomas accounting for more than 90% of the cases (24). Nevertheless, the 5th edition of the WHO classification updated in 2021 only kept the term of IDH wild-type glioblastoma (22). 1p/19q co-deletion, also known as an important prognostic factor, is associated with increased sensitivity to chemotherapy compared with 1p/19q non-co-deleted tumors (25). Additionally, the survival analysis of this study demonstrated that the high expression level of COL5A1 correlated with poor prognosis of glioma patients. In summary, COL5A1 is overexpressed in gliomas prone to malignant progression and can be used as an important marker of tumor malignancy and poor prognosis.

A number of COL5A1 co-expressed genes mentioned in this study have been studied in gliomas. Follistatin-like protein 1 (FSTL1), as one of the positively correlated genes with COL5A1, was reported to promote TMZ resistance by sequentially regulating DIP2A protein distribution, H3K9 acetylation (H3K9Ac), and MGMT transcription (26). In addition, C–X–C motif chemokine receptor 4 (CXCR4), C–X–C motif ligand 3 (CXCL3), and matrix metallopeptidase 7 (MMP7), also considered as positively correlated genes, were reported as oncogenic genes and facilitate growth, invasion, migration, and chemoresistance in gliomas (27–29). On the other hand, aldolase C (ALDOC) and cytochrome oxidase subunit 7A2 (COX7A2), which were both negatively co-expressed genes of COL5A1, were good prognostic factors of gliomas (30, 31). Furthermore, the PPI networks of the co-expressed genes suggested that collagen members, such as COL8A1, COL1A1, COL1A2, and the like, were the main connected components with COL5A1. Prolyl-4-hydroxylase subunit 2 (P4HA2), as one of the hub genes interacted the collagen members above, was reported to play an important role in tumor proliferation, migration, invasion, and epithelial-to-mesenchymal transition (EMT) in gliomas (32, 33). Another similar hub gene Serpin family H member 1 (SERPINH1), also known as HSP47, was proved to promote GBM stem-like cell survival by modulating tumor microenvironment through TGF-β pathway (34) and enhance glioma angiogenesis through HIF1α-VEGFR2 signaling (35).

In this study, the function enrichment analysis based on COL5A1 and its co-expressed genes concluded a number of significant KEGG pathways and GO categories participating in the malignant behavior of proliferation, invasion, and migration in gliomas. For instance, the ECM receptor interaction pathway is considered to play an important role in glioma progression and correlate with tumor invasion (36, 37). The focal adhesion pathway is also famous as an oncogenic pathway in gliomas (38). Focal adhesion kinase (FAK) was reported to regulate migration and invasion of gliomas *in vitro* and *in vivo (* 39). Transforming growth factor beta is a classical tumor progression factor in gliomas (40, 41), and its related pathways or biological processes (e.g., GO: 0032914 positive regulation of transforming growth factor beta 1 production, GO: 0007179 transforming growth factor beta receptor signaling pathway) were enriched in our analysis. In general, GO categories related to regulation of ECM stand out among function enrichment analyses, indicating that COL5A1 promotes glioma progression mainly through organization of ECM or activating ECM receptors.

The specific COL5A1 small interfering RNA was used to exploit the therapeutic potential of inhibiting COL5A1 in gliomas. Downregulation of COL5A1 was proved to inhibit proliferation and migration of glioma cells for the first time in our study. The results of flow cytometry in this study demonstrated that decreased COL5A1 improved the percent of glioma cells at the G1 phase. The G1 arrest of tumor cells was the mechanism of many antitumor drugs in gliomas, such as moxidectin, sinomenine, and ampelopsin (42–44). We also found that downregulation of COL5A1 could sensitize glioma cells’ response to TMZ. Since TMZ was reported to cause senescence and apoptosis in glioblastoma (45), we compared the apoptosis ratios of U87 cells exposed to 50 μg/ml TMZ from three groups and found that inhibition of COL5A1 could enhance apoptosis induced by TMZ significantly. These results suggest that COL5A1 may be a potential therapeutic target in gliomas.

Additionally, we evaluated the effect of COL5A1 on the abundance and distribution of TIICs in gliomas. TIICs are part of a complex microenvironment that promotes and/or regulates tumor development and growth (46). It was known that glioma created a profoundly immunosuppressive environment both locally and systemically, which severely restricted the efficacy of immunotherapy (47). Dendritic cells (DCs) have an integral role in influencing the immune response and are the subset of cells in the tumor microenvironment (TME) to which antitumor T cells are attracted, but they may alter their role from being immunostimulatory to immunosuppressive at different stages of cancer progression (48). TIDCs of gliomas are impaired in a variety of ways, which lead these DCs to confer immune suppression rather than immune stimulation at the local TME (49). In our study, the increasing expression of COL5A1 was positively related to more TIDCs in both LGG and GBM. Meanwhile, high amounts of TIDCs indicated worse prognosis in LGG and GBM, implying that COL5A1 may participate in the formation of immunosuppressive TME in gliomas.

## Conclusion

COL5A1 is overexpressed in gliomas with more malignant features and plays an oncogenic role in tumor cells’ proliferation, migration, and chemoresistance to TMZ. Given its prognostic value, biological functions, and relationship with tumor-infiltrating immune cells, COL5A1 is a reliable biomarker and potential therapeutic target of gliomas.

## Data Availability Statement

The datasets generated and analyzed in the current study are available from the corresponding authors on reasonable request.

## Ethics Statement

The studies involving human participants were reviewed and approved by the Ethics Committee of Union Hospital of Huazhong University of Science and Technology. The patients/participants provided their written informed consent to participate in this study.

## Author Contributions

HZ and PF contributed to the conception and design of the study. ZP and YXW organized the database. SG and YHW performed the statistical analysis. SG wrote the first draft of the manuscript. HZ, PF, XJ, and DL wrote sections of the manuscript. All authors contributed to the manuscript revision and read and approved the submitted version.

## Funding

This work was supported by the Health and Family Planning Commission of Wuhan Municipality (WX17Q42).

## Conflict of Interest

The authors declare that the research was conducted in the absence of any commercial or financial relationships that could be construed as a potential conflict of interest.

## Publisher’s Note

All claims expressed in this article are solely those of the authors and do not necessarily represent those of their affiliated organizations, or those of the publisher, the editors and the reviewers. Any product that may be evaluated in this article, or claim that may be made by its manufacturer, is not guaranteed or endorsed by the publisher.

## References

[1] Ostrom QT Gittleman H Xu J Kromer C Wolinsky Y Kruchko C. CBTRUS Statistical Report: Primary Brain and Other Central Nervous System Tumors Diagnosed in the United States in 2009-2013. Neuro Oncol (2016) 18:v1–v75. doi: 10.1093/neuonc/now207 28475809 PMC8483569

[2] Ostrom QT Bauchet L Davis FG Deltour I Fisher JL Langer CE. The Epidemiology of Glioma in Adults: A "State of the Science" Review. Neuro Oncol (2014) 16:896–913. doi: 10.1093/neuonc/nou087 24842956 PMC4057143

[3] Alonso MM Gomez-Manzano C Bekele BN Yung WK Fueyo J. Adenovirus-Based Strategies Overcome Temozolomide Resistance by Silencing the O6-Methylguanine-DNA Methyltransferase Promoter. Cancer Res (2007) 67:11499–504. doi: 10.1158/0008-5472.CAN-07-5312 18089777

[4] Lee SY. Temozolomide Resistance in Glioblastoma Multiforme. Genes Dis (2016) 3:198–210. doi: 10.1016/j.gendis.2016.04.007 30258889 PMC6150109

[5] Hildebrand KA Frank CB Hart DA. Gene Intervention in Ligament and Tendon: Current Status, Challenges, Future Directions. Gene Ther (2004) 11:368–78. doi: 10.1038/sj.gt.3302198 14724683

[6] Birk DE Fitch JM Babiarz JP Doane KJ Linsenmayer TF. Collagen Fibrillogenesis *In Vitro*: Interaction of Types I and V Collagen Regulates Fibril Diameter. J Cell Sci (1990) 95(Pt 4):649–57. doi: 10.1242/jcs.95.4.649 2384532

[7] Liu W Wei H Gao Z Chen G Liu Y Gao X. COL5A1 may Contribute the Metastasis of Lung Adenocarcinoma. Gene (2018) 665:57–66. doi: 10.1016/j.gene.2018.04.066 29702185

[8] Wei Z Chen L Meng L Han W Huang L Xu A. LncRNA HOTAIR Promotes the Growth and Metastasis of Gastric Cancer by Sponging miR-1277-5p and Upregulating COL5A1. Gastric Cancer (2020) 23:1018–32. doi: 10.1007/s10120-020-01091-3 32583079

[9] Zhao B Song X Guan H. CircACAP2 Promotes Breast Cancer Proliferation and Metastasis by Targeting miR-29a/B-3p-COL5A1 Axis. Life Sci (2020) 244:117179. doi: 10.1016/j.lfs.2019.117179 31863774

[10] Feng G Ma HM Huang HB Li YW Zhang P Huang JJ. Overexpression of COL5A1 Promotes Tumor Progression and Metastasis and Correlates With Poor Survival of Patients With Clear Cell Renal Cell Carcinoma. Cancer Manag Res (2019) 11:1263–74. doi: 10.2147/CMAR.S188216 PMC6369854 30799953

[11] Zhang J Zhang J Wang F Xu X Li X Guan W. Overexpressed COL5A1 is Correlated With Tumor Progression, Paclitaxel Resistance, and Tumor-Infiltrating Immune Cells in Ovarian Cancer. J Cell Physiol (2021) 236(10):6907–19. doi: 10.1002/jcp.30350 33655494

[12] Goldman MJ Craft B Hastie M Repečka K McDade F Kamath A. Visualizing and Interpreting Cancer Genomics Data *via* the Xena Platform. Nat Biotechnol (2020) 38:675–8. doi: 10.1038/s41587-020-0546-8 PMC7386072 32444850

[13] Zhao Z Zhang KN Wang Q Li G Zeng F Zhang Y. Chinese Glioma Genome Atlas (CGGA): A Comprehensive Resource With Functional Genomic Data From Chinese Glioma Patients. Genomics Proteomics Bioinf (2021) 19(1):1–12. doi: 10.1101/2020.01.20.911982 PMC8498921 33662628

[14] Vasaikar SV Straub P Wang J Zhang B. LinkedOmics: Analyzing Multi-Omics Data Within and Across 32 Cancer Types. Nucleic Acids Res (2018) 46:D956–d963. doi: 10.1093/nar/gkx1090 29136207 PMC5753188

[15] Huang da W Sherman BT Lempicki RA. Systematic and Integrative Analysis of Large Gene Lists Using DAVID Bioinformatics Resources. Nat Protoc (2009) 4:44–57. doi: 10.1038/nprot.2008.211 19131956

[16] Li T Fan J Wang B Traugh N Chen Q Liu JS. TIMER: A Web Server for Comprehensive Analysis of Tumor-Infiltrating Immune Cells. Cancer Res (2017) 77:e108–10. doi: 10.1158/0008-5472.CAN-17-0307 PMC6042652 29092952

[17] Ricard-Blum S. The Collagen Family. Cold Spring Harb Perspect Biol (2011) 3:a004978. doi: 10.1101/cshperspect.a004978 21421911 PMC3003457

[18] Xu S Xu H Wang W Li S Li H Li T. The Role of Collagen in Cancer: From Bench to Bedside. J Transl Med (2019) 17:309. doi: 10.1186/s12967-019-2058-1 31521169 PMC6744664

[19] Fu X Zhang P Song H Wu C Li S Li S. LTBP1 Plays a Potential Bridge Between Depressive Disorder and Glioblastoma. J Transl Med (2020) 18:391. doi: 10.1186/s12967-020-02509-3 33059753 PMC7566028

[20] Deng T Gong YZ Wang XK Liao XW Huang KT Zhu GZ. Use of Genome-Scale Integrated Analysis to Identify Key Genes and Potential Molecular Mechanisms in Recurrence of Lower-Grade Brain Glioma. Med Sci Monit (2019) 25:3716–27. doi: 10.12659/MSM.913602 PMC6537664 31104065

[21] Louis DN Perry A Reifenberger G von Deimling A Figarella-Branger D Cavenee WK. The 2016 World Health Organization Classification of Tumors of the Central Nervous System: A Summary. Acta Neuropathol (2016) 131:803–20. doi: 10.1007/s00401-016-1545-1 27157931

[22] Louis DN Perry A Wesseling P Brat DJ Cree IA Figarella-Branger D. The 2021 WHO Classification of Tumors of the Central Nervous System: A Summary. Neuro Oncol (2021) 23:1231–51. doi: 10.1093/neuonc/noab106 PMC8328013 34185076

[23] Aibaidula A Chan AK Shi Z Li Y Zhang R Yang R. Adult IDH Wild-Type Lower-Grade Gliomas Should be Further Stratified. Neuro Oncol (2017) 19:1327–37. doi: 10.1093/neuonc/nox078 PMC5596181 28575485

[24] Le Rhun E Preusser M Roth P Reardon DA van den Bent M Wen P. Molecular Targeted Therapy of Glioblastoma. Cancer Treat Rev (2019) 80:101896. doi: 10.1016/j.ctrv.2019.101896 31541850

[25] van den Bent MJ Baumert B Erridge SC Vogelbaum MA Nowak AK Sanson M. Interim Results From the CATNON Trial (EORTC Study 26053-22054) of Treatment With Concurrent and Adjuvant Temozolomide for 1p/19q non-Co-Deleted Anaplastic Glioma: A Phase 3, Randomised, Open-Label Intergroup Study. Lancet (2017) 390:1645–53. doi: 10.1016/S0140-6736(17)31442-3 PMC5806535 28801186

[26] Nie E Miao F Jin X Wu W Zhou X Zeng A. Fstl1/DIP2A/MGMT Signaling Pathway Plays Important Roles in Temozolomide Resistance in Glioblastoma. Oncogene (2019) 38:2706–21. doi: 10.1038/s41388-018-0596-2 PMC6484760 30542120

[27] Yi L Zhou X Li T Liu P Hai L Tong L. Notch1 Signaling Pathway Promotes Invasion, Self-Renewal and Growth of Glioma Initiating Cells *via* Modulating Chemokine System CXCL12/CXCR4. J Exp Clin Cancer Res (2019) 38:339. doi: 10.1186/s13046-019-1319-4 31382985 PMC6683584

[28] Bruyère C Mijatovic T Lonez C Spiegl-Kreinecker S Berger W Kast RE. Temozolomide-Induced Modification of the CXC Chemokine Network in Experimental Gliomas. Int J Oncol (2011) 38:1453–64. doi: 10.3892/ijo.2011.964 21399866

[29] Wang Z Wang L Liang Z Xi Y. Long Non-Coding RNA BCAR4 Promotes Growth, Invasion and Tumorigenicity by Targeting miR-2276 to Upregulate MMP7 Expression in Glioma. Onco Targets Ther (2019) 12:10963–73. doi: 10.2147/OTT.S226026 PMC6913310 31849498

[30] Chang YC Tsai HF Huang SP Chen CL Hsiao M Tsai WC. Enrichment of Aldolase C Correlates With Low Non-Mutated IDH1 Expression and Predicts a Favorable Prognosis in Glioblastomas. Cancers (Basel) (2019) 11(9):1238. doi: 10.3390/cancers11091238 PMC6770576 31450822

[31] Deng S Li Y Yi G Lei B Guo M Xiang W. Overexpression of COX7A2 is Associated With a Good Prognosis in Patients With Glioma. J Neurooncol (2018) 136:41–50. doi: 10.1007/s11060-017-2637-z 29079956

[32] Wu Y Zhang X Wang J Ji R Zhang L Qin J. P4HA2 Promotes Cell Proliferation and Migration in Glioblastoma. Oncol Lett (2021) 22:601. doi: 10.3892/ol.2021.12862 34188703 PMC8228437

[33] Lin J Jiang L Wang X Wei W Song C Cui Y. P4HA2 Promotes Epithelial-To-Mesenchymal Transition and Glioma Malignancy Through the Collagen-Dependent PI3K/AKT Pathway. J Oncol (2021) 2021:1406853. doi: 10.1155/2021/1406853 34434233 PMC8382519

[34] Jiang X Zhou T Wang Z Qi B Xia H. HSP47 Promotes Glioblastoma Stemlike Cell Survival by Modulating Tumor Microenvironment Extracellular Matrix Through TGF-β Pathway. ACS Chem Neurosci (2017) 8:128–34. doi: 10.1021/acschemneuro.6b00253 27696866

[35] Wu ZB Cai L Lin SJ Leng ZG Guo YH Yang WL. Heat Shock Protein 47 Promotes Glioma Angiogenesis. Brain Pathol (2016) 26:31–42. doi: 10.1111/bpa.12256 25758142 PMC8029092

[36] Shevchenko V Arnotskaya N Pak O Sharma A Sharma HS Khotimchenko Y. Molecular Determinants of the Interaction Between Glioblastoma CD133(+) Cancer Stem Cells and the Extracellular Matrix. Int Rev Neurobiol (2020) 151:155–69. doi: 10.1016/bs.irn.2020.03.005 32448605

[37] Jiang Y He J Guo Y Tao H Pu F Li Y. Identification of Genes Related to Low-Grade Glioma Progression and Prognosis Based on Integrated Transcriptome Analysis. J Cell Biochem (2020) 121:3099–111. doi: 10.1002/jcb.29577 31886582

[38] Venkatesh HS Tam LT Woo PJ Lennon J Nagaraja S Gillespie SM. Targeting Neuronal Activity-Regulated Neuroligin-3 Dependency in High-Grade Glioma. Nature (2017) 549:533–7. doi: 10.1038/nature24014 PMC5891832 28959975

[39] Liu TJ LaFortune T Honda T Ohmori O Hatakeyama S Meyer T. Inhibition of Both Focal Adhesion Kinase and Insulin-Like Growth Factor-I Receptor Kinase Suppresses Glioma Proliferation *In Vitro* and *In Vivo* . Mol Cancer Ther (2007) 6:1357–67. doi: 10.1158/1535-7163.MCT-06-0476 17431114

[40] Pan YB Zhang CH Wang SQ Ai PH Chen K Zhu L. Transforming Growth Factor Beta Induced (TGFBI) Is a Potential Signature Gene for Mesenchymal Subtype High-Grade Glioma. J Neurooncol (2018) 137:395–407. doi: 10.1007/s11060-017-2729-9 29294230

[41] Jennings MT Pietenpol JA. The Role of Transforming Growth Factor Beta in Glioma Progression. J Neurooncol (1998) 36:123–40. doi: 10.1023/A:1005863419880 9525812

[42] Song D Liang H Qu B Li Y Liu J Chen C. Moxidectin Inhibits Glioma Cell Viability by Inducing G0/G1 Cell Cycle Arrest and Apoptosis. Oncol Rep (2018) 40:1348–58. doi: 10.3892/or.2018.6561 PMC6072399 30015956

[43] He X Maimaiti M Jiao Y Meng X Li H. Sinomenine Induces G1-Phase Cell Cycle Arrest and Apoptosis in Malignant Glioma Cells *via* Downregulation of Sirtuin 1 and Induction of P53 Acetylation. Technol Cancer Res Treat (2018) 17:1533034618770305. doi: 10.1177/1533034618770305 29756546 PMC5952277

[44] Guo Z Guozhang H Wang H Li Z Liu N. Ampelopsin Inhibits Human Glioma Through Inducing Apoptosis and Autophagy Dependent on ROS Generation and JNK Pathway. BioMed Pharmacother (2019) 116:108524. doi: 10.1016/j.biopha.2018.12.136 31108349

[45] Günther W Pawlak E Damasceno R Arnold H Terzis AJ. Temozolomide Induces Apoptosis and Senescence in Glioma Cells Cultured as Multicellular Spheroids. Br J Cancer (2003) 88:463–9. doi: 10.1038/sj.bjc.6600711 PMC2747547 12569392

[46] Domingues P González-Tablas M Otero Á Pascual D Miranda D Ruiz L. Tumor Infiltrating Immune Cells in Gliomas and Meningiomas. Brain Behav Immun (2016) 53:1–15. doi: 10.1016/j.bbi.2015.07.019 26216710

[47] Grabowski MM Sankey EW Ryan KJ Chongsathidkiet P Lorrey SJ Wilkinson DS. Immune Suppression in Gliomas. J Neurooncol (2021) 151:3–12. doi: 10.1007/s11060-020-03483-y 32542437 PMC7843555

[48] Engelhardt JJ Boldajipour B Beemiller P Pandurangi P Sorensen C Werb Z. Marginating Dendritic Cells of the Tumor Microenvironment Cross-Present Tumor Antigens and Stably Engage Tumor-Specific T Cells. Cancer Cell (2012) 21:402–17. doi: 10.1016/j.ccr.2012.01.008 PMC3311997 22439936

[49] Tran Janco JM Lamichhane P Karyampudi L Knutson KL. Tumor-Infiltrating Dendritic Cells in Cancer Pathogenesis. J Immunol (2015) 194:2985–91. doi: 10.4049/jimmunol.1403134 PMC4369768 25795789

